# Use of Computing Devices as Sensors to Measure Their Impact on Primary and Secondary Students’ Performance

**DOI:** 10.3390/s19143226

**Published:** 2019-07-22

**Authors:** Francisco Luis Fernández-Soriano, Belén López, Raquel Martínez-España, Andrés Muñoz, Magdalena Cantabella

**Affiliations:** Escuela Politécnica, Universidad Católica de Murcia, 30107 Guadalupe, Spain

**Keywords:** computing devices, primary and secondary education, students’ performance, Learning Management System, learning analytics

## Abstract

The constant innovation in new technologies and the increase in the use of computing devices in different areas of the society have contributed to a digital transformation in almost every sector. This digital transformation has also reached the world of education, making it possible for members of the educational community to adopt Learning Management Systems (LMS), where the digital contents replacing the traditional textbooks are exploited and managed. This article aims to study the relationship between the type of computing device from which students access the LMS and how affects their performance. To achieve this, the LMS accesses of students in a school comprising from elementary to bachelor’s degree stages have been monitored by means of different computing devices acting as sensors to gather data such as the type of device and operating system used by the students.The main conclusion is that students who access the LMS improve significantly their performance and that the type of device and the operating system has an influence in the number of passed subjects. Moreover, a predictive model has been generated to predict the number of passed subjects according to these factors, showing promising results.

## 1. Introduction

As a consequence of the accelerated advance of digital transformation in today’s society, different sectors and services have notably improved in their efficiency, sustainability and innovation processes. This is the case of the sectors of education, health, transport and farming, among others. This change has meant a whole digital revolution, transforming the fundamental nature of organizations, which highlights the need to update their digital processes [1].

In the last decade the Internet of Things (IoT) brings us closer to an increasingly transparent integration between the real and computing worlds. The digitization of processes in different social sectors has been enhanced by the continued proliferation and impact that have led to the use of mobile devices in society. This fact has enabled institutions to base their workflows through software systems controlled by digital devices [2,3]. These factors have generated a special interest in the educational community, which use Learning Management Systems (LMS) as the main tool for managing their academic content. As a consequence, both students and teachers use LMS on a daily frequency to access learning resources through different digital devices, such as computers, tablets or mobile phones [4]. In this context it may be of special interest to evaluate how the teaching and learning processes would improve depending on the type of device from which the access is performed [5].

A solution adopted in recent years for obtaining the data generated from digital devices is the integration of sensors, used to analyze the usability and benefits obtained from new emerging technologies, which allow us to quantify accurately the data obtained through digital devices in different areas [6,7,8,9]. In the area of education, the use of sensors as measurement tools in various educational disciplines is noteworthy. For example, the use of inertial sensors to monitor and evaluate movement patterns of different sports activities in schools, with the aim of engaging students in sport disciplines [10].

The use of sensors is one of the potential lines in digital transformation, industry 4.0 and a growing field in the education sector. The use of sensors offers new learning opportunities, allows the acceptance of e-learning materials to be measured and creates new learning strategies for lecturers and students [11,12]. For example, the use of Virtual Technologies in Education will allow immersion experiences interacting with objects, concepts or processes, developing new methodologies adaptable to any educational level, from primary school to higher education [13]. Another aspect of interest is to quantify the benefit of the use of mobile devices in the classroom through the sensors that they integrate, with the purpose of evaluating the academic performance of students and narrowing the generational technological gap by adopting new educational methodologies [14,15]. Another research line is the manufacture of a type of paper equipped with electromagnetic sensors, which could interact with electronic devices, and whose main destination would be the education field [16]. In [17] it is proposed a fog computing model to improve the limitations of LMS based on the analysis of the streams of data collected from students’ smartphones (GPS location and timestamp data) and heart rate bands while they are connected to the LMS. The main improvements aim at obtaining real-time prediction of students’ performances and fatigues and uncovering new didactic models according to location and times of study.

The use of mobile devices in the classroom initially generates rejection in the lecturers, since they are perceived as a distraction in the classroom. The disorders associated with the use of this technology have even been included as an addiction, generating a debate among the scientific community as to whether or not these devices should be excluded from the educational system [18]. However, it is important to progress towards a digital conversion in teaching methodologies and to take advantage of the benefits offered by digital tools [19,20]. Thanks to the use of new technologies, new opportunities are created in the education sector that challenge traditional methodologies. The regular use of digital technology and devices in classrooms has a positive effect on both digital skills and attitudes towards technology, which can benefit the learning process of students [21,22]. Therefore, it is a great challenge for schools to be able to incorporate and adapt this digital revolution to their classrooms [23,24].

The results obtained in different works demonstrate that thanks to the use of new technologies in the classroom, access to materials in the classroom will improve, both for lecturers and students, as they can access learning resources at anytime and anyplace. Another factor that favors the use of technology in the classroom is the improvement of communication and feedback between students, lecturers and parents. The use of technology allows students to grow as an educational community, extending beyond the walls of the classroom. As a result, it may bring students closer to multiple resources and through different perspectives, as well as to new educational levels and challenges not otherwise available [25,26].

It has also been investigated the impact of mobile devices on different educational stages, concluding that the use of such devices have become an essential learning tool with great potential both inside and outside the classroom. It reveals an innovative and motivating educational process for the students that allows them to be an active part of it. At the same time, in general, the use of mobile devices in education is better than when desktop computers are used or no mobile devices are used at all in the teaching and learning process [27,28,29]. One of the problems detected for e-learning is the access to materials and activities from mobile devices. It is necessary to adequate learning environments and develop new applications that allow to personalize and adapt digital content to digital devices and thus remove barriers and improve the quality of teaching, as discussed elsewhere [30,31,32].

It is essential to implement methodological changes in the teaching materials in order to adapt these devices. The integration of these technologies should be gradual and transparent from the earliest stages, with the aim of minimizing the digital barrier in the period of higher education [33,34]. It is important to adapt the use of new technologies taking into account the factors included in the Model of Acceptance of Technology and Use of Technology, so in this way the digital devices will have a greater acceptance by students and lecturers as a new learning tool [35,36]. Therefore, it is necessary to study new strategies for digital instruction, which help lecturers to promote the use of new teaching technologies that benefit teaching and learning processes, in order to improve the academic results of students.

The main objective of this work is to analyze the impact on the students’ performance according to the use of computing devices for connecting to the LMS. These computing devices act as sensors to obtain different data such as the type of accessing device (e.g., computer, tablet, phablet or smartphone), the type of operating system or the time when the student logs in the LMS. By means of this analysis it is expected to identify which are the most optimal patterns of connection to the LMS and be able to demonstrate that the students’ performance can be conditioned by different factors such as the type of mobile device from which the student connects, operating system used and the frequency of connections, among others.

The rest of paper is structured as follows. The methodology followed in this work and the data utilized are explained in Section 2. Section 3 shows the results obtained in the case study and these are discussed in Section 4. Finally, Section 5 summarizes the findings obtained in this work and shows possible future lines of research.

## 2. Material and Methods

This section describes the experimentation research method applied in this work following the guidelines given in the book “Basic of software engineering experimentation” [37]. First, the research problem is defined through several research questions addressing the topics considered in the experiment (Section 2.1). Then, the design of the experiment is explained (Section 2.2) as well as the specific techniques used to perform the experiment (Section 2.3).

### 2.1. Research Problem

The aim of this experimental research is to measure the impact on the student’s performance depending on the use of different computing devices, considering these devices as sensors measuring the student’s activity in the LMS. In order to reach the main objective of this work, the following research questions (RQ) have been defined:

- RQ1. Are there differences in the number of passed subjects between students who connect to the LMS and those who do not connect?
- RQ2. Are there differences in the number of failed subjects between students who connect to the LMS and those who do not connect?
- RQ3. For those students who do connect to the LMS, are there differences in the number of passed subjects depending on the computing device being used?
- RQ4. For those students who do connect to the LMS, are there differences in the number of failed subjects depending on the computing device being used?
- RQ5. For those students who do connect to the LMS, are there differences in the number of passed subjects depending on the operating system being used?
- RQ6. For those students who do connect to the LMS, are there differences in the number of failed subjects depending on the operating system being used?
- RQ7. Is it possible to generate a predictive model on the number of passed subjects taking into account the data from the computing device (e.g., type of device and OS)?

Observe that we separately study the possible effect of the use of devices and operating system (OS) on the student’s performance. The reason for this separation is that there is not a unique OS for each type of device (e.g., Windows, iOS and Android for tablets), and therefore we cannot assume that they are dependent variables.

### 2.2. Constructing the Experiment

The experiment proposed in this paper has been performed in the Santo Domingo School network, which is composed of several school centers in Alicante, Spain. This institution offers a whole educational plan from pre-school to high school. The majority of the students’ families have an upper-middle socioeconomic status. For this study the stages taken into consideration start from Fourth Grade (9-to-10-year-old students) to the last year of the bachelor’s degree (17-to-18-year-old students). All the students in this stage are allowed to use computing devices (desktop or mobile) to connect to the LMS. In this manner, the computing devices work as a type of sensor to collect data from students every time they are connected to the LMS. It is important to note that students are not restricted to any kind of device and that they can connect to the LMS anytime and anyplace. Likewise, the use of a specific type of device (i.e., smartphone, tablet, PC, etc.) does not imply the students use the same OS for that type of device.

The data related to the study correspond to the academic year 2017/18 and they have been gathered from two sources: (1) the student’s file for gender, number of passed subjects and number of failed subjects in the whole academic year; (2) the logs for the student’s connections composed of the timestamp (in the format “dd-mm-yyyy hh:mm:ss”), device type and operating system. From these logs we have also obtained the following data per each student: Total number of logins in the LMS in the whole academic year, average number of logins per week, maximum and minimum number of logins per week, most frequent connection day and most frequent connection time slot divided into hours (0–23). Other variables such as family problems (divorced parents and/or problems with siblings) or financial problems (related to the payment of school fees) have been considered to verify their relationship to the student’s performance. However, the preliminary results do not show a significant relationship between the student’s performance and the possible economic and/or family problems, so these variables have not been included in this study.

A stratified sampling method has been used in the first place to construct the experiment. We have grouped the students’ data following the Spanish education system, namely *Elementary* (in this study comprising Fourth, Fifth and Sixth grades), *Secondary* (four years) and *Bachelor’s Degree* (two years). The number of participants in each group has been calculated in proportion to the total number of students in those stages. Then, for each group a clustering sampling method has been employed to divide students between those that have ever used a computing device to connect to the LMS and those who have never connected. Finally, a simple random sampling has been applied to select student for each cluster in each stage.

It is worth mentioning that a control group cannot be created for this study due to legal reasons raised by the Santo Domingo School. To solve this problem, students were volunteered to participate in the experiment by ensuring with the school faculty that the contents to be evaluated were the same for those students who did not want to participate in the study. Likewise, there has been no homogeneous distribution of device types among students as this would oblige them to purchase a specific device. The solution adopted has been to allow the use of any type of device included in the study or to borrow a device already available in the school. For this study specifically the school lent 192 iPads (tablets).

### 2.3. Conducting the Experiment

A total of 1,938 students have been analysed, out of which 881 (45.45%) are men and 1057 (54.55%) are women. From the total number of students, 1011 students (52.17%) have ever connected to the LMS, whereas 927 students (47.83%) have never been logged on. Table 1 shows the distribution of the sample according to the education stage.

Table 2 shows the number of students using each device and operating system to connect to the LMS. In general, the most used OS in computers is Windows, iOS in tablets and Android and iOS in smartphones. Note that there are two students using the Android OS in computers in the Bachelor’s degree stage. This is due to the fact that these two students are using a Smart TV box device with an Android OS according to the recorded logs. We have considered this kind of device as “Desktop Computer” since it is normally fixed in a room. It is also noteworthy that 12 students in this stage and other 3 in secondary are using an iOS emulator in their PC. We have asked them about this and they stated that they wanted to use some apps for iOS that were recommended by some teachers in their own PC at the same time they accessed to the LMS.

The analysis of the data has been carried out in three steps. Firstly, a descriptive analysis is performed on the variables available from the connection logs (type of devices and OS). Secondly, an inferential analysis is done to answer RQ1-RQ6. The Kolmogorov-Smirnov normality test is employed to examine if the variables are normally distributed. For the analysis the Mann-Withney test and the Kruskal-Wallis test with Bonferroni adjustment have been used to compare two means and more than two means, respectively [38]. In all the tests we consider the *p*-value < 0.05 as significant. Finally, predictive tests are performed to evaluate RQ7. To this end, the M5Rules algorithm is applied [39]. In a nutshell, this technique generates a list of decision trees for regression problems using the separate-and-conquer technique. It returns a list of rules to calculate a value for the output variable according to the answers received for each input variable.

The SPSS 24.0 software has been employed to execute the two first stages and Weka suite for the third one.

## 3. Results

In this section we present the results obtained for the study proposed in Section 2. First of all, the results of the descriptive analysis on the sensor (computing device) variables are shown (see Section 3.1). Then, the results of the inferential analysis on these variables related to the students’ performance are described (see Section 3.2). Finally, the results of applying the M5 rules technique to predict the number of subjects passed by the students according to their LMS connection behavior are shown (see Section 3.3).

### 3.1. Descriptive Statistics

Initially we analyze the data from a global point of view, without discerning the different educational stages. Table 3 shows the maximum, mean and standard deviation (SD) of the number of passed and failed subjects of students who have ever logged on the LMS and who have never logged on it. Observe that the total number of subjects is 9 in elementary, 13 in secondary and 10 in Bachelor’s degree. This Table 3 shows that students who have ever logged on have an average of 9.27 passed subjects and 1.1 failed subjects with a standard deviation of 2.26 and 1.75, respectively. In addition, the maximum number of failed subjects is 8, whereas the maximum number of passed subjects is 13. The maximum number of connections registered for a single student is 498 logins, whereas the average number of logins per student is 135.2 (SD 88.76). Students who have never logged on to the LMS have an average of 7.65 passed subjects and 2.25 failed subjects, with a standard deviation of 2.8 and 2.64, respectively. In addition, the maximum number of passed subjects by a student without connection to the system is 13 and the maximum number of failed subjects is 12.

Now the analysis is divided into the different educational stages. Table 4 shows for each of the educational stages, the number of failed subjects and the number of passed subjects taking into account whether or not the student has ever logged into the LMS. The maximum number of connections registered for a single student is 415 logins for elementary, 498 for secondary and 372 for Bachelor’s degree, whereas the average number of logins per student is 138.44 (SD 84.43) for elementary, 153.39 (SD 94.10) for secondary and 96.61 (SD 67.87) for Bachelor’s degree.

Table 5 shows for each of the educational stages and for students who have ever logged in the LMS, the number of passed and failed subjects considering the type of device used to log in to the LMS. The frequency column shows the number of students using a device for a particular stage, and in brackets is the percentage that the use of that device represents in the total of students who have ever connected for that stage. Note that “Phablets” are used by very few students so their results are not conclusive. In the bachelor’s education stage, the average number of passed subjects (8.02) is higher when students use the tablet as a connecting device. The tablet is also the connecting device which students in this stage obtain fewer failed subjects on average (1.30 failed subjects). For the secondary education stage, students logged into the LMS using the computer have on average more subjects passed (10.82). The students who on average get fewer failed subjects (1.06) are those logged using tablets. For the elementary stage, the results are very similar for any device, although the students connected by means of the smartphones obtain on average a better result in the number of passed subjects.

Table 6 shows the number of failed and passed subjects for students in each of the three educational stages and types of operating systems used to log into the LMS. The frequency column shows the number of students using a type of operating system for a particular stage, and in brackets is the percentage the operating system represents of the total for that stage. In general, students using Windows as the operating system have a greater average number of passed subjects and a lesser average number of failed ones for all stages. Observe that the ChromeOS operating system is only used by students in the secondary stage.

### 3.2. Inferential Statistics

This section shows the results of the different statistical tests for RQ1-RQ6. First, we must study whether the data allow the use of parametric or non-parametric tests according to the distribution of the variables. The Kolmogorov-Smirnov normality test returns a 0.0 *p*-value for the variables representing both the number of passed subject and the number of failed subjects. Then, the non-parametric tests will be used for the hypothesis defined in the research questions.

**Figure 1:**
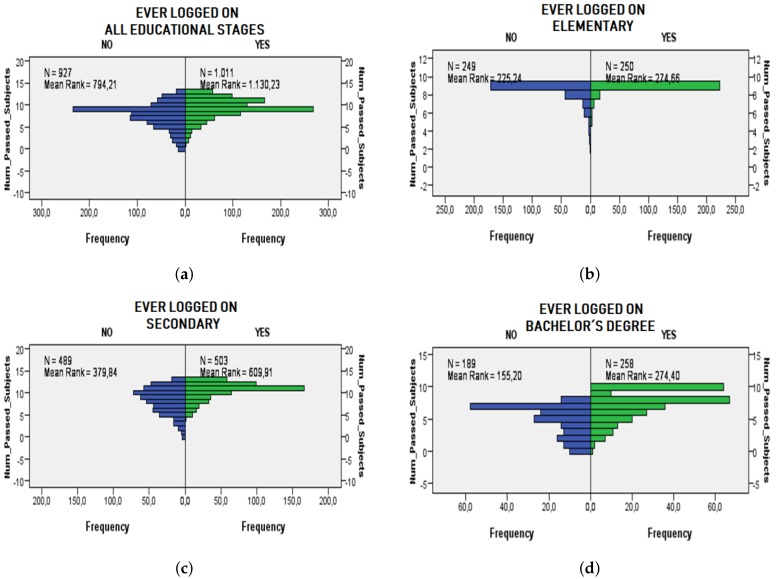
Statistical differences according to the Mann-Whitney test considering if there is any relation between accessing or not accessing the LMS and the number of passed subjects. The right side of the subfigures (in green color) represents the students who have logged into the LMS and the left side (blue color) represents the students who have not logged into the LMS. (**a**) Differences between the number of passed subjects for students accessing and not accessing the LMS considering all the educational stages. (**b**) Differences between the number of passed subjects for students accessing and not accessing the LMS considering the elementary stage. (**c**) Differences between the number of passed subjects for students accessing and not accessing the LMS considering the secondary stage. (**d**) Differences between the number of passed subjects for students accessing and not accessing the LMS considering the bachelor degree’s stage.

The Mann-Whitney test is performed to evaluate RQ1, namely if there are significant differences between the number of subjects passed between students who have ever logged into the LMS and those who have not. Four tests have been performed considering all the education stages at the same time (Figure 1a), elementary (Figure 1b), secondary (Figure 1c) and Bachelor’s degree (Figure 1d) stages. The *p*-value obtained for the four tests is 0.0, concluding that there are significant differences with a 95% confidence level in the number of subjects passed between students who have ever logged into the LMS and those who have not. Figure 1 shows the significant differences in graphical form and also indicates the sample (N) and the mean “Mean Rank” of each test.

**Figure 2:**
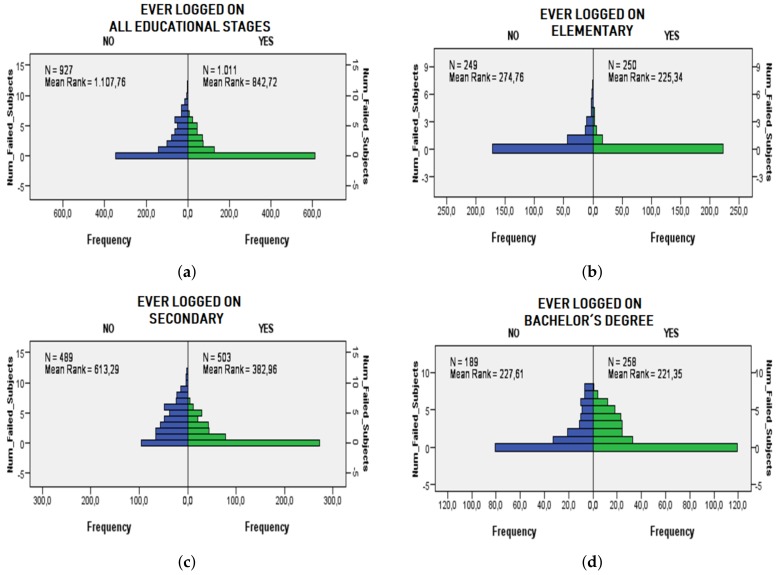
Statistical differences according to the Mann-Whitney test considering if there is any relation between accessing or not accessing the LMS and the number of failed subjects. The right side of the subfigures (in green color) represents the students who have logged into the LMS and the left side (blue color) represents the students who have not logged into the LMS. (**a**) Differences between the number of failed subjects for students accessing and not accessing the LMS considering all the educational stages. (**b**) Differences between the number of failed subjects for students accessing and not accessing the LMS considering the elementary stage. (**c**) Differences between the number of failed subjects for students accessing and not accessing the LMS considering the secondary stage. (**d**) Differences between the number of failed subjects for students accessing and not accessing the LMS considering the bachelor degree’s stage.

Similarly, RQ2 is evaluated through the Mann-Whitney test as well to demonstrate if there are significant differences between the number of subjects failed between students who have ever logged into the LMS and those who have not. Four tests have been performed considering all the education stages (Figure 2a), elementary (Figure 2b), secondary (Figure 2c) and Bachelor’s degree (Figure 2d) stages. The results indicate a *p*-value value of 0.0 for the three first tests, showing that there are significant differences, with a 95% confidence level, of the number of failed subjects for students who ever connect to the LMS and those who do not connect for the elementary and secondary stages. Contrarily, for the bachelor’s degree stage the *p*-value is 0.5, thus there are no significant differences for this stage related to RQ2.

The RQ3 is evaluated by means of the Kruskal-Wallis test to compare samples looking for significant differences in the number of passed subjects depending on the type of computing device used when connecting to the LMS. The *p*-value obtained is equal to 0.0 indicating that there are significant differences with a 95% confidence level between the number of passed subjects and the type of device used by the student. By analyzing the *p*-values for each pair of device type and then adjusting the *p*-value using the Bonferroni test, there are significant differences with a 95% confidence level between smartphones and PCs, between smartphones and tablets, and between PCs and tablets. These *p*-values are shown in Table 7, where the last column (“Adj. *P*-value”) shows the *p*-value adjusted by Bonferroni’s post-hoc test.

The RQ4 follows RQ3 but taking failed subjects into account instead. Table 8 shows the results of the Kruskal-Wallis test with the *p*-value obtained for each pair of compared devices adjusted with Bonferroni’s post-hoc test. There are significant differences with a 95% confidence level for the number of failed subjects when comparing the use between tablets and PCs, between smartphones and tablets and between smartphones and PCs.

Finally, RQ5 and RQ6 are evaluated through the Kruskal-Wallis test for differences between the number of passed/failed subjects and the operating system used by students to connect to the LMS.

Table 9 shows the values for RQ5. The adjusted *p*-values of this table indicate that there are differences in the number of passed subjects when comparing the use of Windows, MacOS and Android; when comparing Android with iOS, MacOS and ChromeOS; and when comparing iOS and ChromeOS.

Likewise, Table 10 shows the values for RQ6. The adjusted *p*-values of this table indicate that there are differences in the number of failed subjects when comparing the use of Windows with MacOS and iOs and when comparing the use of Android with MacOs and iOS.

### 3.3. Predicting the Number of Passed Subjects Based on Computing Device Data

The last result to be shown is related to RQ7, namely the generation of a predictive model to predict the number of passed subjects using data from the computing devices. To carry out the creation of this initial predictive model, we have first created a dataset composed of the following attributes:

- Gender
- Educational stage
- Type of device
- Type of operating system
- Total number of connections during the academic year
- Average number of connections per week
- Maximum number of connections per week
- Minimum number of connections per week
- Most frequent connection day
- Most frequent connection time slot
- Number of passed subjects

The first ten variables are input attributes and the last variable “number of passed subjects” is the target attribute. The technique used both to create the model and to classify its fitness has been the M5Rules algorithm, described in Section 2.3. One advantage of this technique is the readability of the results, including easy-to-understand explanations about them. The experiment has been performed by dividing the original dataset into two: 80% has been used for the training phase and 20% has been used to test the model. To ensure the robustness of the model, instead of performing a cross validation, we have chosen to repeat the experiment 5 times, taking different sets of training and test each time. Thus, if the results obtained each time do not vary, we can affirm that the model is stable and robust. The measures used to measure the model fitness are the root mean squared error (RMSE), the mean absolute error (MAE) and the correlation coefficient (CC) between the input attributes and the output variable. In addition, each one of these measures has associated the standard deviation (SD) value that has been obtained from the 5 repetitions. Table 11 shows the mean results of the models created along with their standard deviation.

In total the model creates 206 rules. The following two rules are an example of standard rules created by the M5Rules algorithm to determine the number of passed subjects per student. The variable E.Stage indicates the educational stage by referring code 1 to “Elementary” and code 3 to “Bachelor’s degree”. The other variables involved are OSystem, CodDevice, MaxconnectionsWeek, NconnectionsWeek and NpassedSubject that represent the variables type of operating system, Type of device, maximum number of connections per week, average number of connections per week and number of passed subjects respectively.

IF (E.Stage=3)∧(OSystem=5)∧(CodDevice=0)∧(MaxconnectionsWeek>=7.245)∧(NconnectionsWeek>4.63)

THEN NpassedSubject=10.4463

IF (E.Stage=1)∧(CodDevice=1)(MaxconnectionsWeek<=3.845)∧(NconnectionsWeek<2.29)

THEN NpassedSubject=6.3052

In addition to the prediction model, we have also obtained a selection of the most relevant variables for the creation of the model. We consider a relevant variable as the one that has been used in at least 90% of the rules obtained in the predictive model. The most relevant variables are (in brackets the percentage of occurrence of each variable regarding the total number of rules): Average number of connections per week (92.3%), maximum number of connections per week (95.4%), educational stage and type of device (98.1%).

## 4. Discussion

The descriptive statistics applied on the data set indicate that, on average, students who log into the LMS obtain a better average of passed and failed subjects, even taking into account the standard deviation of these figures. Thus, on average, students with connections to the system pass almost two more subjects and fail one subject less than students who never connect. The most common devices used to log into the LMS are tablets, followed by PCs and smartphones. Regarding the operating system, the most used is iOS followed by Windows and Android. It must be taken into account that phablets are rarely used and their data are not representative. After consulting the school about the reason for the low use of this device, it is concluded that it is not effective in terms of performance and cost.

The analysis of the proposed RQs leads us to the following conclusions:

- RQ1. There are significant differences between the number of subjects passed by a student with respect to whether or not the student has ever logged into the LMS. Analyzing the values in Table 4 along with the statistical test demonstrates that a student passes more subjects if he or she ever logged into the LMS. This conclusion holds considering each educational stage separately.
- RQ2. There are significant differences between the number of subjects failed by a student with respect to whether or not the student has ever logged into the LMS. Analyzing the values in Table 4 along with the statistical test it is concluded that a student fails fewer subjects if he or she ever logs into the LMS. This conclusion holds considering each educational stage separately except for the bachelor’s degree stage, where there is no significant evidence among the relation of students who have ever accessed the LMS and the number of failed subjects.
- RQ3. There are significant differences between the number of subjects passed by a student and the type of device used to log into the LMS. Specifically, and studying in depth the results of the statistical test and Table 5, there is a greater number of subjects passed for students who use tablets, followed by students who use PCs and finally for students who use smartphones.
- RQ4. There are significant differences between the number of subjects failed by a student and the type of device used to log into the LMS. Analyzing the results of the statistical test and Table 5, there are fewer failed subjects for students who use tablets, followed by students who use PCs and finally students who use smartphones.
- RQ5. There are significant differences between the number of subjects passed by a student and the operating system used to log into the LMS. Studying the results of the statistical test and Table 6, the students who use the MacOS and ChromeOS obtain a greater number of passed subjects, followed by the students who use the iOS operating system, then the students who use the Windows operating, and finally the students who use Android.
- RQ6. There are significant differences between the number of subjects failed by a student and the operating system used to log into the LMS. In this case, students using MacOS, iOS and ChromeOS obtain a similar number of failed subjects without significant differences. This number of failed subjects is lower than students using Windows and Android.
- RQ7. The initial model of rules created using the M5Rules algorithm is robust since after repeating it for 5 times randomly it has given similar results obtaining a low standard deviation. Although the results can be improved, the model obtains an average error of almost 2 subjects when predicting the number of passed subjects and obtains a correlation between the input attributes and the target attribute of 72% on average. There have been variables not included in this first initial model as the time slot or the most frequent day of connection. These variables will be analyzed in a more complex model that can provide more adjusted results.

We can therefore conclude that the use of computing devices at anytime for studying positively influences students in the educational stages of elementary, secondary and bachelor’s degree. It both reduces the number of failed subjects and increases the number of passed subjects.

## 5. Conclusions and Future Work

The main objective of this work resides in analyzing the impact on the students’ performance when connecting to an LMS (Learning Management System) through different digital devices (PCs, smartphones, tablets and phablets). These devices act as sensors for collecting data about the students’ habits in their access to the LMS, taking into account variables such as the type of device, operating system and number of connections per student. Three educational stages have been considered in this study, namely elementary, secondary and bachelor’s degree.

The results of this work show that students who connect to the LMS pass a greater number of subjects in all stages. In addition, and taking into account the limitations of the study as explained below, the use of a specific computing device may help to obtain a better education performance. Thus, the number of passed subjects increases for students who use tablets as the preferred computing device. It has been also shown that the operating system used is an influential factor in the number of passed subjects, since students using MacOS get the best results. Moreover, a rule model has been built to predict the number of passed subjects considering the number of connections to the LMS and the type of device as the most relevant factors.

It should be highlighted the relevance of the operating system as a factor on the students’ performance according to our study. In this sense, there are several studies in this line showing that aspects such as the usability and the interface design of the OS build a greater engagement for the students [40,41]. The operating systems taken into account in this study (Windows, Chrome OS, Android, iOS and MacOS) can integrate a variety of e-learning applications. In this line, the findings of this study show that students who use MacOS as operating system have a better success rate on average. As a result, the school associated with this study will be recommending as the main option those devices that allow the use of such an operating system.

There are some limitations on this study. One of the most relevant is that the results obtained may be partially conditioned due to the use of tablets as the most used device, since it is the device recommended by the school. Another limitation factor is the notable use of Ipads, in this case due to the school’s loan policy. For this study, the school lent 192 Ipads, almost 19% of the devices used in the study. However, we think that the rest of devices and OS are well represented in the sample except for Phablets and the Chrome OS, which can be considered as marginal elements in the study.

Future works in this research line include the study of data in more school centres and educational stages. New variables will also be considered to help identify new patterns of behaviour, such as the duration of the connection and the specific academic term. Moreover, an extension and improvement of the predictive model will be conducted to improve not only the prediction of the number of passed subjects but also to find a pattern of the students’ behavior based on their intermediate grades in the same academic year. Finally, it would be interesting to integrate new sensors such as cameras that can provide more information on the students’ behaviour and emotions.

## Figures and Tables

**Table 1:** Number of students involved in the study grouped by education stage, including gender information and the number of students who have ever logged on.

Education Stage	# of Students	Gender	Ever Logged on
Elementary	499	M: 262 (52.5%) F: 237 (47.5%)	Y: 250 (50%) N: 249 (50%)
Secondary	992	M: 463 (46.7%) F: 529 (53.3%)	Y: 503 (51%) N: 489 (49%)
Bachelor’s degree	447	M: 156 (34.9%) F: 291 (65.1%)	Y: 258 (58.8%) N: 189 (41.2%)

**Table 2:** Number of students per device and operating system disaggregated by different educational stages (Bachelor’s degree, Secondary and Elementary).

Educational Stage	Device	OS	*N* of Students
**Bachelor’s degree**	Computer	Android	2
		iOS	12
		macOS	29
		Windows	88
	Smartphone	Android	30
		iOS	42
	Tablet	Android	2
		iOS	51
	Phablets	Android	2
**Secondary**	Computer	Chrome OS	17
		iOS	3
		macOS	32
		Windows	30
	Smartphone	Android	17
		iOS	13
	Tablet	Android	11
		iOS	378
	Phablets	Android	2
**Elementary**	Computer	macOS	1
		Windows	15
	Smartphone	Android	8
		iOS	2
	Tablet	Android	2
		iOS	221
	Phablets	Android	1

**Table 3:** Maximum, mean and standard deviation (SD) of the number of subjects failed and passed by students of all stages considering whether they have ever connected to the LMS.

Ever Logged on	Attributes	Max	Mean	SD
**YES**	***Num_Failed_Subjects***	8	1.1	1.75
	***Num_Passed_Subjects***	13	9.27	2.26
**NO**	***Num_Failed_Subjects***	12	2.29	2.64
	***Num_Passed_Subjects***	13	7.65	2.8

**Table 4:** Descriptive analysis of the number of subjects failed and passed by students divided by educational stages.

Educational Stage	Ever Logged on	Attributes	Max	Mean	SD
**Bachelor’s degree**	**YES**	***Num_Failed_Subjects***	8	**1.72**	2.064
		***Num_Passed_Subjects***	10	**7.29**	2.298
	**NO**	***Num_Failed_Subjects***	8	**1.89**	2.390
		***Num_Passed_Subjects***	8	**4.96**	2.363
**Secondary**	**YES**	***Num_Failed_Subjects***	7	**1.24**	1.766
		***Num_Passed_Subjects***	13	**10.51**	1.910
	**NO**	***Num_Failed_Subjects***	12	**3.30**	2.790
		***Num_Passed_Subjects***	13	**8.31**	2.910
**Elementary**	**YES**	***Num_Failed_Subjects***	4	**0.20**	0.651
		***Num_Passed_Subjects***	9	**8.80**	0.651
	**NO**	***Num_Failed_Subjects***	7	**0.60**	1.188
		***Num_Passed_Subjects***	9	**8.40**	1.188

**Table 5:** Number of students, percentage, maximum, mean and standard deviation of number of passed and failed subject for students who have ever logged in the LMS according to the educational stage and connecting device. The best results considering the mean number of subjects passed for each educational stage are highlighted in bold.

Educational Stage	Device	N. of Students (%)	Attributes	Max	Mean	SD
**Bachelor’s degree**	**Computer**	131 (50.8%)	Num_Failed_Subjects	7	1.51	1.951
			Num_Passed_Subjects	10	7.42	2.201
	**Smartphone**	72 (27.9%)	Num_Failed_Subjects	8	2.39	2.323
			Num_Passed_Subjects	10	6.53	2.584
	**Tablet**	53 (20.5%)	Num_Failed_Subjects	5	1.30	1.761
			Num_Passed_Subjects	10	**8.02**	1.855
	**Phablets**	2 (0.8%)	Num_Failed_Subjects	4	2.00	2.828
			Num_Passed_Subjects	8	7.00	1.414
**Secondary**	**Computer**	82 (16.3%)	Num_Failed_Subjects	7	1.55	2.044
			Num_Passed_Subjects	13	**10.82**	2.363
	**Smartphone**	30 (6.0%)	Num_Failed_Subjects	7	2.80	2.265
			Num_Passed_Subjects	12	8.50	2.271
	**Tablet**	389 (77.3%)	Num_Failed_Subjects	6	1.06	1.592
			Num_Passed_Subjects	13	10.60	1.681
	**Phablets**	2 (0.4%)	Num_Failed_Subjects	2	1.00	1.414
			Num_Passed_Subjects	11	**11.00**	0.0
**Elementary**	**Computer**	16 (6.4%)	Num_Failed_Subjects	4	0.31	1.014
			Num_Passed_Subjects	9	8.69	1.014
	**Smartphone**	10 (4.0%)	Num_Failed_Subjects	0	0.0	0.0
			Num_Passed_Subjects	9	9.00	0.0
	**Tablet**	223 (89.2%)	Num_Failed_Subjects	4	0.2	0.634
			Num_Passed_Subjects	9	**8.80**	0.634
	**Phablets**	1 (0.4%)	Num_Failed_Subjects	0	0.0	0.0
			Num_Passed_Subjects	9	9.00	0.0

**Table 6:** Number of students, percentage, maximum, mean and standard deviation of number of passed and failed subjects for students who have ever logged in the LMS according to the educational stage and operating system used for the connection. The best results considering the mean number of subjects passed for each educational stageare highlighted in bold

Educational Stage	OS	N. of Students (%)	Attributes	Max	Mean	SD
**Bachelor’s degree**	**Android**	36 (14.0%)	Num_Failed_Subjects	8	2.19	2.505
			Num_Passed_Subjects	10	6.86	2.587
	**iOS**	105(40.7%)	Num_Failed_Subjects	6	1.61	1.959
			Num_Passed_Subjects	10	7.42	2.227
	**macOS**	29(11.2%)	Num_Failed_Subjects	6	1.14	1.726
			Num_Passed_Subjects	10	**7.83**	2.172
	**Windows**	88(34.1%)	Num_Failed_Subjects	7	1.84	2.067
			Num_Passed_Subjects	10	7.14	2.290
**Secondary**	**Android**	30(6.0%)	Num_Failed_Subjects	6	2.27	2.116
			Num_Passed_Subjects	12	9.07	2.149
	**Chrome OS**	17(3.4%)	Num_Failed_Subjects	5	1.71	1.724
			Num_Passed_Subjects	13	11.29	1.724
	**iOS**	394(78.3%)	Num_Failed_Subjects	7	1.11	1.649
			Num_Passed_Subjects	13	10.55	1.747
	**macOS**	32(6.4%)	Num_Failed_Subjects	5	0.69	1.378
			Num_Passed_Subjects	13	**12.13**	1.581
	**Windows**	30(6.0%)	Num_Failed_Subjects	7	2.27	2.477
			Num_Passed_Subjects	13	9.40	2.486
**Elementary**	**Android**	11(4.4%)	Num_Failed_Subjects	3	0.27	0.905
			Num_Passed_Subjects	9	8.73	0.905
	**iOS**	223(89.2)	Num_Failed_Subjects	4	0.18	0.606
			Num_Passed_Subjects	9	**8.82**	0.606
	**macOS**	1(0.4%)	Num_Failed_Subjects	0	0.0	0.0
			Num_Passed_Subjects	9	9.00	0.0
	**Windows**	15(6.0%)	Num_Failed_Subjects	4	0.33	1.047
			Num_Passed_Subjects	9	8.67	1.047

**Table 7:** Values of the Kruskal-Wallis statistical test and the Bonferroni post-hoc test related to the differences between the number of passed subjects and the type of device used to access the LMS.

Device1-Device2	Test Statistics	Std. Error	Std. Test Statistics	*P*-Value	Adj. *p*-Value
**Smartphone - Computer**	153.14	33.19	4.61	0.00	0.00
**Smartphone - Tablet**	−281.443	29.40	−9.57	0.00	0.00
**Computer - Tablet**	−128.29	22.05	−5.81	0.00	0.00

**Table 8:** Values of the Kruskal-Wallis statistical test and the Bonferroni post-hoc test related to the differences between the number of failed subjects and the type of device used to access the LMS.

Device1- Device2	Test Statistics	Std. Error	Std. Test Statistics	*p*-Value	Adj. *p*-Value
**Tablet - Computer**	95.41	19.685	4.84	0.00	0.00
**Tablet - Smartphone**	195.34	26.24	7.44	0.00	0.00
**Computer - Smartphone**	−99.93	29.62	−3.37	0.01	0.04

**Table 9:** Values of the Kruskal-Wallis statistical test and the Bonferroni post-hoc test showing significant differences between the number of passed subjects and the OS used for connections.

OS1 - OS2	Test Statistics	Std. Error	Std. Test Statistics	*p*-Value	Adj. *p*-Value
**Windows - iOS**	207.11	27.16	7.62	0.00	0.00
**Windows - macOS**	272.01	44.26	6.14	0.00	0.00
**Windows - Chrome OS**	439.01	74.14	5.92	0.00	0.00
**Android - iOS**	−184.96	34.51	−5.36	0.00	0.00
**Android - macOS**	−249.86	49.11	−5.08	0.00	0.00
**Android - Chrome OS**	−416.85	77.13	−5.41	0.00	0.00
**iOS - Chrome OS**	231.899	70.63	3.28	0.01	0.10

**Table 10:** Values of the Kruskal-Wallis statistical test and the Bonferroni post-hoc test showing significant differences between the number of failed subjects and the OS used for connections.

OS1 - OS2	Test Statistics	Std. Error	Std. Test Statistics	*p*-value	Adj. *p*-value
**iOS - Windows**	−120.72	24.24	−4.98	0.00	0.00
**iOS - Android**	130.697	30.80	4.24	0.00	0.00
**macOS - Windows**	−114.68	39.51	−2.90	0.04	0.04
**macOS - Android**	124.66	43.85	2.84	0.04	0.05

**Table 11:** Mean of evaluation results of the M5Rules model after five repetitions to predict the number of passed subjects.

	CC	MAE	RMSE
**Mean**	0.7279	1.3718	1.86967
**SD**	0.0116	0.0311	0.0446
